# Anterior coracoid coverage and the glenoid roof morphometry: a radiographic model of directional shoulder instability

**DOI:** 10.1016/j.jseint.2026.101777

**Published:** 2026-08-05

**Authors:** Giuseppe Bonsignore, William G. Blakeney, Abdelkader Shekhbihi, Igor Shirinskiy, Dirk Maier, Andreas Marc Müller, Stefan Bauer

**Affiliations:** a Department of Orthopaedic and Trauma Surgery, St. Josefs Hospital Wiesbaden, Wiesbaden, Germany; b Centre de Chirurgie de l'Épaule, du Coude et de la Main, Service d'Orthopédie et Traumatologie, EHC, Morges, Vaud, Switzerland; c Division of Surgery, Medical School, University of Western Australia, Perth, Western Australia, Australia; d Department of Orthopaedics, Royal Perth Hospital, Perth, Western Australia, Australia; e Department of Orthopaedic and Trauma Surgery, University Hospital Basel (USB), Basel, Switzerland; f University Hospital Basel (USB), Surgical Outcome Research Centre (SORC), Basel, Switzerland

**Keywords:** Shoulder instability, Glenoid roof coverage, Coracoid, Acromion morphology, Radiographic measurement, Neer Y-view

## Abstract

**Background:**

Quantification of anterior and posterior bony containment of the glenoid roof provides a reproducible radiographic framework for characterizing direction-specific shoulder instability on standard radiographs. However, simple and reproducible radiographic parameters integrating anterior and posterior roof morphology have not been systematically defined. The aim of this study was to compare posterior acromial coverage (PAC), anterior coracoid coverage (ACC), and glenoid roof coverage, defined as the sum of ACC and PAC, among shoulders with neutral alignment, static posterior subluxation (Walch B), and chronic anterior instability before Latarjet surgery. We hypothesized that PAC would be reduced in static posterior subluxation (Walch B), whereas ACC would be reduced in chronic anterior instability compared with neutral shoulders.

**Methods:**

A prospectively maintained institutional shoulder registry was retrospectively reviewed to identify 150 standardized pre-operative Neer lateral Y-view radiographs, including 50 clinically stable arthritic shoulders with neutral glenoid alignment (Neutral), 50 shoulders with static posterior subluxation (Walch B), and 50 shoulders with chronic anterior instability treated with a Latarjet procedure (Latarjet). PAC and ACC were measured independently by 2 observers using predefined radiographic landmarks. Between-group comparisons were performed using Mann–Whitney *U* tests. Diagnostic performance was assessed using receiver operating characteristic analysis, and associations with group allocation were explored using logistic regression. Interobserver reliability was evaluated using intraclass correlation coefficients.

**Results:**

PAC was lower in Walch B than in Neutral (63.2° [55.3-68.9] vs. 70.0° [65.9-77.9]; *P* < .001) and discriminated Walch B from Neutral (area under the curve 0.76; 95% CI 0.66-0.85). ACC was lower in Latarjet than in Neutral (70.1° [61.2-77.1] vs. 75.6° [69.6-83.7]; *P* = .001) and moderately discriminated Latarjet from Neutral (area under the curve 0.70; 95% CI 0.59-0.79). Glenoid roof coverage was highest in Neutral and reduced in Walch B (*P* = .006) and Latarjet (*P* < .001). Interobserver reliability was excellent for all parameters (intraclass correlation coefficient ≥0.85).

**Conclusion:**

PAC and ACC quantify direction-specific alterations of superior glenoid roof morphology and provide a reproducible radiographic framework for identifying reduced anterior or posterior humeral head coverage on standardized Y-view radiographs.

The shoulder is the most mobile joint in the human body, and this mobility reduces intrinsic stability. Glenohumeral stability depends on the interaction between osseous morphology and soft-tissue restraints.^1^ Traumatic and recurrent anterior or posterior instability is common, particularly in young and active patients. Several morphologic concepts have been proposed to explain direction-specific instability, including glenoid bone loss, the glenoid track, and humeral head defects. While these concepts are clinically useful, they do not provide a simple geometric explanation for direction-specific instability. However, recent morphometric and biomechanical studies suggest that variations in acromial and coracoid anatomy influence bony containment and humeral head translation through altered scapular morphology and muscle force vectors.^7^^,^^11^^,^^12^^,^^14^^,^^17^^,^^19^

The superior shoulder suspensory complex as described by Goss forms an osteoligamentous ring between the scapula and clavicle and integrates the variations of coracoid and acromial anatomy.^9^ The roof of this arch thus contributes to bony containment of the glenohumeral joint, but its morphology has so far been described mainly in qualitative terms.

Previous radiographic parameters, including the coracoacromial shoulder arch angle, acromial slope, and coracoacromial arch curvature, have improved characterization of superior shoulder morphology. However, these parameters primarily describe the overall geometry of the coracoacromial arch and do not separately quantify the anterior coracoid and posterior acromial contributions to direction-specific glenohumeral bony containment.^4^^,^^11^^,^^15^, ^16^, ^17^

In addition, no single geometric index currently quantifies the total width of the superior glenoid roof by integrating anterior and posterior coverage into 1 measurable parameter. This gap is clinically relevant. Coracoid-based procedures such as the Trillat or Latarjet increase anterior bony and soft-tissue restraint.^2^^,^^3^^,^^20^^,^^21^ Acromial and glenoid osteotomies address posterior deficiency in shoulders with posterior decentering.^8^^,^^10^ In clinical practice, surgeons frequently rely on initial plain radiographs prior to advanced three-dimensional imaging when deciding between soft-tissue stabilization and bony procedures. Simple and reproducible radiographic indices that quantify anterior and posterior roof coverage may therefore help identify direction-specific bony deficiency and may contribute to surgical assessment.

We therefore introduce anterior coracoid coverage (ACC) and glenoid roof coverage (GRC) as quantitative radiographic descriptors of superior glenoid roof geometry on standardized Neer lateral Y-view radiographs. ACC quantifies anterior glenoid roof coverage, whereas posterior acromial coverage (PAC) reflects posterior bony containment. GRC integrates PAC and ACC into a single angular measure of total glenoid roof coverage. GRC integrates PAC and ACC into a single angular measure of total GRC. While PAC has been previously described, ACC and the integrated GRC have not, to our knowledge, been defined as radiographic parameters.^4^^,^^6^^,^^10^^,^^12^^,^^15^^,^^18^^,^^19^

The aims of this study were as follows: (1) to compare PAC between neutral shoulders (Neutral), serving as a clinically stable reference group, and those with static posterior subluxation (Walch B); (2) to compare ACC between neutral shoulders and those with chronic anterior instability prior to Latarjet surgery (Latarjet); and (3) to assess differences in GRC across these groups.

We hypothesized that shoulders with static posterior subluxation (Walch B) would demonstrate decreased PAC, whereas shoulders with chronic anterior instability would demonstrate decreased ACC. We further hypothesized that GRC would differ among the 3 groups, reflecting distinct patterns of directional scapular coverage.

## Materials and methods

This comparative morphometric study was based on a retrospective analysis of prospectively maintained institutional arthroplasty and instability registries and included 150 shoulders from 150 patients who underwent standardized Neer lateral Y-view radiography between 2017 and 2025.

A target of 50 shoulders per group (Neutral, Walch B, Latarjet) was predefined to allow balanced between-group comparisons and to provide sufficient events per variable for multivariable modeling.

All radiographic examinations were performed according to a standardized institutional shoulder protocol by trained radiographers. The study was conducted in accordance with the Declaration of Helsinki and was approved by the regional ethics committee (CER-VD), Project-ID 2025-02019. All patients had provided pre-operative consent for the use of anonymized data.

### Study population

Three surgically treated cohorts with distinct glenohumeral pathologies were included.

The Latarjet cohort comprised patients with chronic traumatic anterior instability subsequently treated with a Latarjet procedure. For all cohorts, standardized pre-operative anteroposterior and Neer lateral Y-view radiographs were obtained before arthroplasty or Latarjet surgery. Additional cross-sectional imaging consisted of computed tomography (CT) in the degenerative cohorts (Neutral, Walch B) and magnetic resonance imaging in the instability cohort (Latarjet). The 3 groups were selected to represent neutral, posteriorly decentered, and anteriorly unstable morphologies. Neer lateral Y-views were screened in chronological order, and shoulders meeting the clinical and imaging eligibility criteria were enrolled until 50 shoulders per group had been included.

Based on surgical indication and pre-operative imaging, shoulders were categorized into 3 groups:

(1) Neutral,
(2) Walch B, and
(3) Latarjet.

Classification of Walch B morphology followed the original criteria,^22^ including a posterior wear pattern, increased glenoid retroversion, and posterior humeral head subluxation (PHHS).

Neutral glenoids were defined as having glenoid retroversion <10° on pre-operative CT; in this subgroup, median PHHS was 54.0% (Table I). As these patients underwent shoulder arthroplasty for primary glenohumeral osteoarthritis, the Neutral cohort should be interpreted as a clinically stable arthritic reference group rather than a representation of normal scapular morphology. For Neutral and Walch B glenoids, glenoid version, PHHS, and the Walch classification were obtained from pre-operative CT scans and calculated automatically using the Blueprint planning software (version 4.2.2; Stryker, Portage, Michigan, USA).^22^

**Table I:** Demographics and instability profile by cohort.

Variable	Neutral (n = 50)	Walch B (n = 50)	Latarjet (n = 50)
Age, yr - mean ± SD	74.1 ± 7.5	72.5 ± 10.2	27.4 ± 5.7
Age range, yr	54-90	45-88	21-44
Male, n	11	23	42
Female, n	39	27	8
Posterior humeral head subluxation, % - median (IQR)∗	54.0 (46.8-62.8)	71.0 (67.0-77.0)	-
Glenoid retroversion, ° - median (IQR)∗	4.0 (3.0-7.0)	11.0 (9.0-16.0)	-
Anterior dislocations, n - median (IQR)†	-	-	4 (3-6)

*PHHS*, posterior humeral head subluxation; *IQR*, interquartile range; *CT*, computed tomography; *SD*, standard deviation.

∗ PHHS and glenoid retroversion were assessed only in the arthroplasty cohorts (Neutral and Walch B) based on pre-operative CT.

† Anterior dislocations were prospectively documented in the Latarjet instability cohort; in the Neutral and Walch B cohorts no anterior shoulder instability was recorded.

Pre-operative magnetic resonance imaging was reviewed for bony and soft-tissue Bankart lesions, labral pathology, and Hill–Sachs defects. Demographic data (age, sex), number of anterior dislocations, and generalized hyperlaxity (Beighton score^5^) were recorded.

Patients in the Neutral and Walch B groups underwent shoulder arthroplasty. Patients in the instability cohort underwent open Latarjet procedures. All operations were performed by a fellowship-trained senior shoulder surgeon. Inclusion criteria were an age of >20 years and the availability of standardized pre-operative anteroposterior and Neer lateral Y-view radiographs. Exclusion criteria were multidirectional instability, connective tissue disorders, documented skeletal dysplasia, prior bony augmentation procedures, incomplete cross-sectional imaging, or inadequate Y-view quality. Radiographs with insufficient visualization of the acromial or coracoid contour or with rotational malalignment were excluded. A total of 18 shoulders were excluded based on these criteria. This study conforms to the STROBE guidelines.

### Radiographic evaluation

Standardized pre-operative Neer lateral Y-view radiographs were obtained with patients in a standing position and the arm in neutral rotation according to a uniform institutional shoulder protocol. Adequacy of projection was verified by 2 fellowship-trained shoulder surgeons.

Radiographs were screened for adequacy prior to inclusion according to predefined quality criteria consistent with those described by Meyer et al.^17^

A radiograph was considered suitable if:

(1) The coracoid process and scapular spine formed symmetric upper limbs of a well-defined scapular Y configuration, indicating a true lateral projection,
(2) The humeral head did not overlap the supraspinatus outlet,
(3) Approximately 45-55% of the humeral head projected superior to the glenoid center, and
(4) The subacromial space was clearly visible, indicating appropriate scapular rotation. Radiographs not meeting all criteria were excluded.

All measurements were performed digitally using Telemis Medical PACS (version 4.97; Telemis SA, Louvain-la-Neuve, Belgium) according to a predefined standardized protocol. Observers were blinded to clinical outcomes at the time of measurement.

#### Glenoid center and reference axis

The glenoid center was defined as the best-fit focal intersection of the sclerotic cortical condensations representing the scapular spine pillar, coracoid pillar, and scapular body pillar on the Neer lateral Y-view. This intersection corresponds to the structural convergence of the 3 scapular components and served as the origin for all angular and linear measurements.

The vertical scapular reference axis was established by drawing a best-fit line along the longitudinal axis of the scapular blade through its midportion and passing through the glenoid center. This axis represented the anatomic vertical orientation of the scapular body and served as the common reference for all angular measurements.

#### Angular parameters

**Figure 1:**
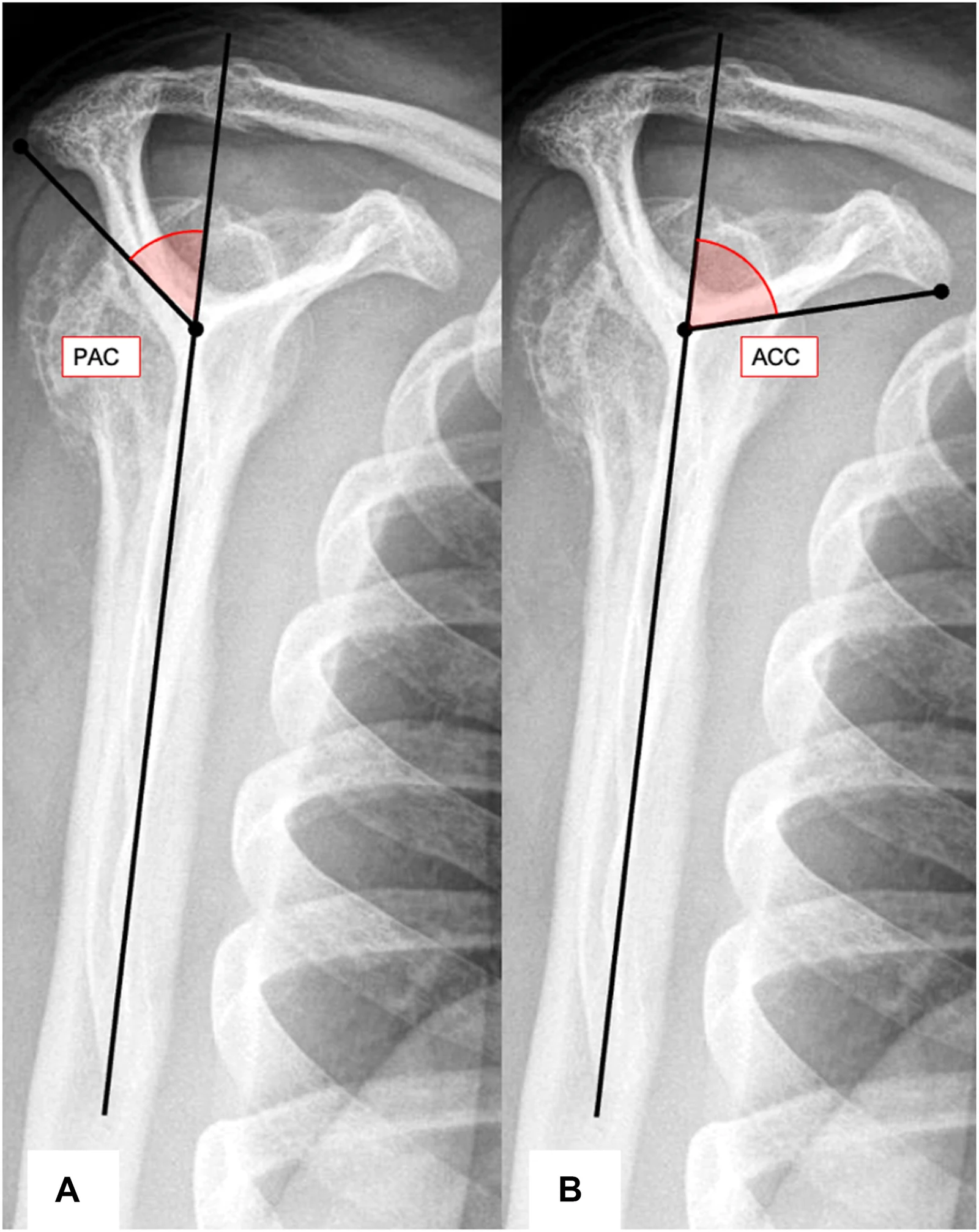
Measurement of PAC and ACC on standardized Neer lateral Y-view radiographs. (**A**) Posterior acromial coverage (PAC) was defined as the angle between the vertical scapular reference axis and a line drawn from the glenoid center to the most posterior-inferior cortical intersection point of the acromion. (**B**) Anterior coracoid coverage (ACC) was defined as the angle between the same vertical scapular reference axis and a line drawn from the glenoid center to the most inferior cortical point of the coracoid process. The glenoid center was defined as the best-fit intersection of the sclerotic cortical condensations of the scapular spine, coracoid, and scapular body.

PAC was defined as the angle between the vertical scapular reference axis and a line connecting the glenoid center to the most posterior-inferior cortical point of the acromion (Fig. 1*A*).

ACC was defined analogously as the angle between the vertical scapular reference axis and a line connecting the glenoid center to the most inferior cortical point of the coracoid process (Fig. 1*B*). Both angles were referenced to the same vertical scapular axis.

**Figure 2:**
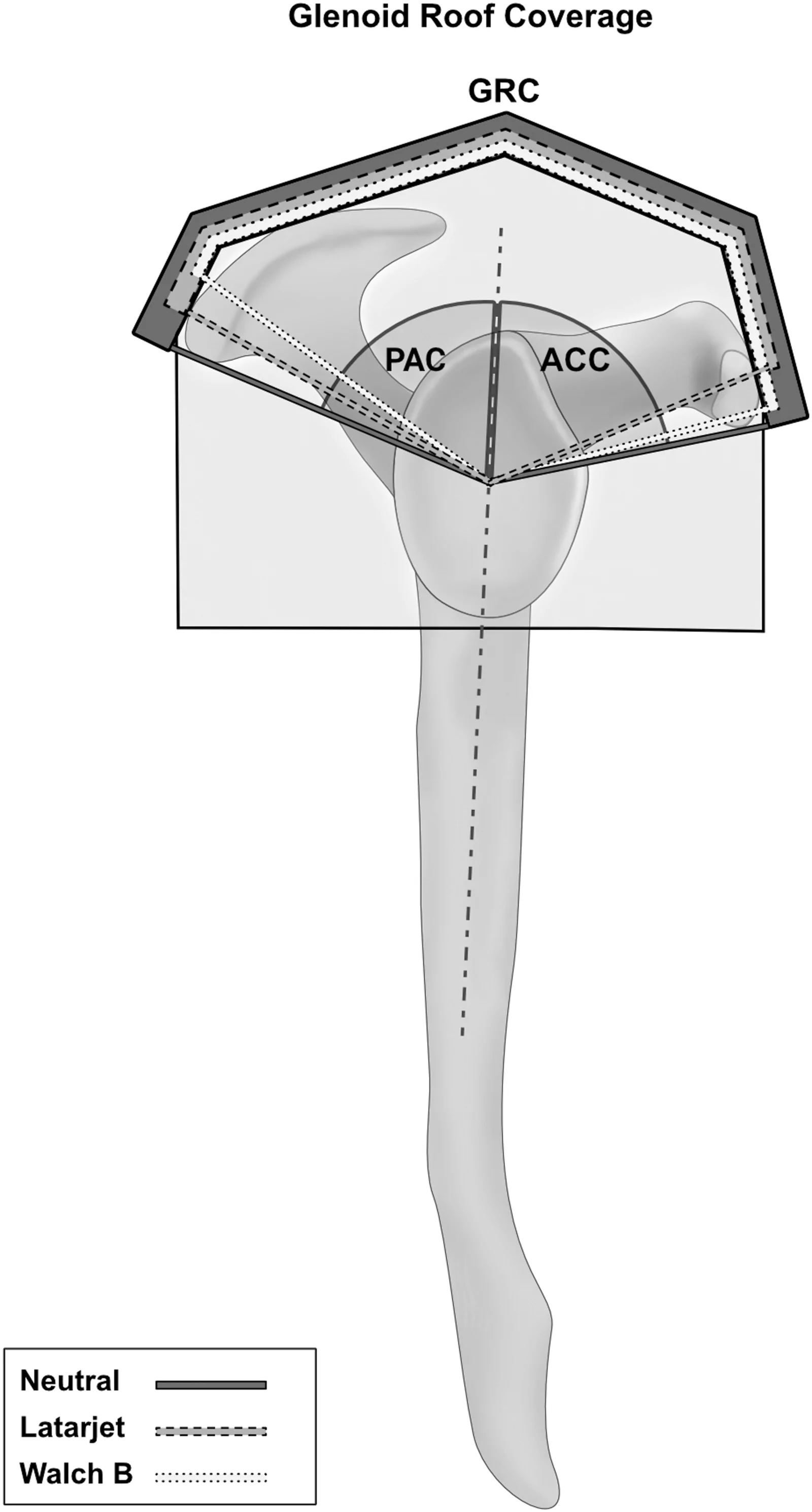
Glenoid roof model illustrating PAC, ACC, and GRC. In shoulders with anterior instability (Latarjet), the glenoid roof demonstrates reduced anterior coverage, reflected by lower anterior coracoid coverage (ACC). In shoulders with posterior instability (Walch B), posterior roof coverage is reduced, reflected by lower posterior acromial coverage (PAC). *GRC*, glenoid roof coverage.

GRC was calculated as the sum of PAC and ACC and represents the continuous angular span of the superior glenoid roof (Fig. 2).

#### Vertical parameters

**Figure 3:**
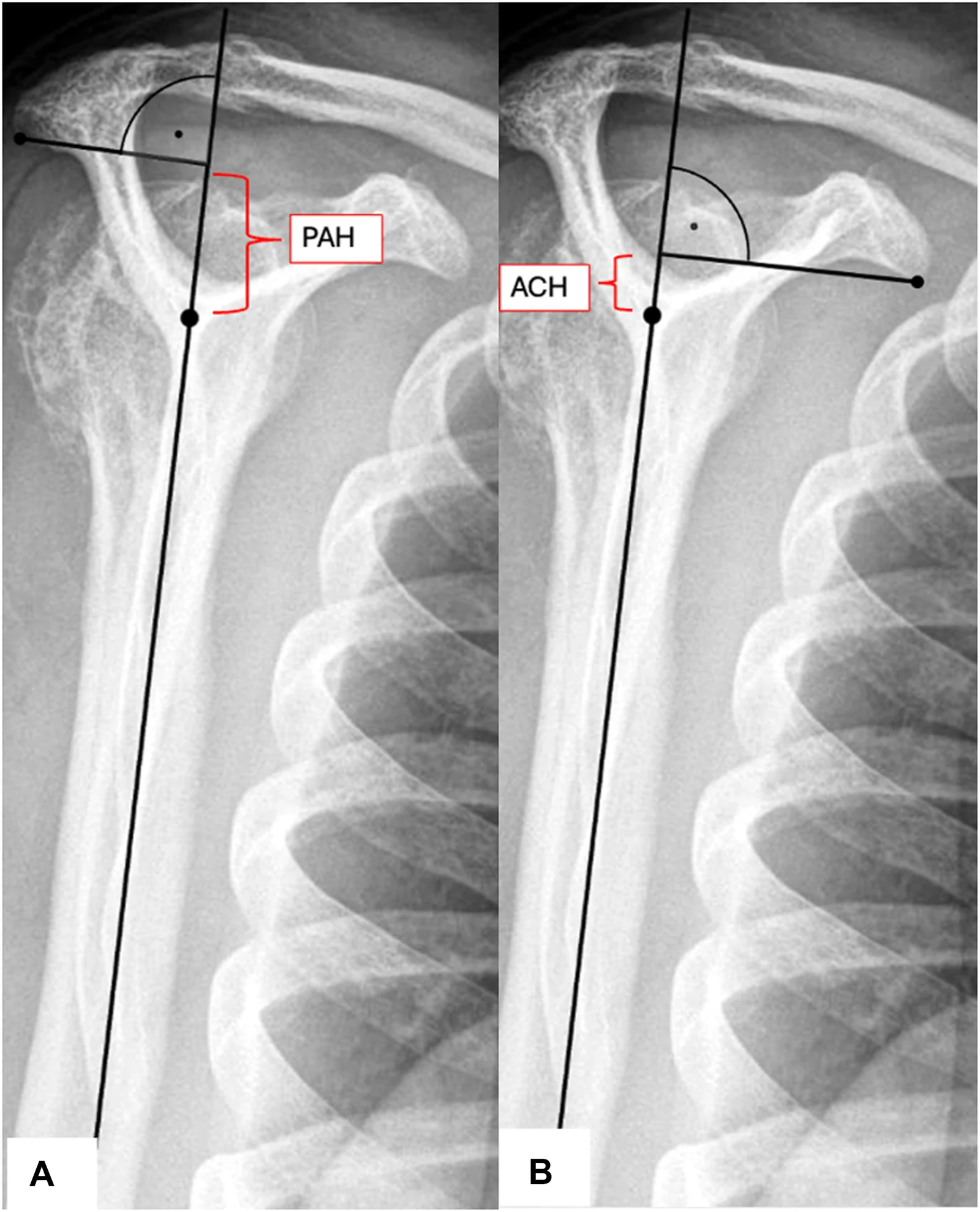
Vertical glenoid roof measurements on standardized Neer lateral Y-view radiographs. (**A**) Posterior acromial height (PAH) was defined as the perpendicular distance from the glenoid *Center* to the most posterior-inferior cortical point of the acromion, measured orthogonally to the vertical scapular reference axis. (**B**) Anterior coracoid height (ACH) was defined as the perpendicular distance from the glenoid *Center* to the most inferior cortical point of the coracoid, measured orthogonally to the same reference axis.

Posterior acromial height (PAH) was defined as the perpendicular distance from the glenoid center to the most posterior-inferior cortical point of the acromion, measured orthogonally to the vertical scapular reference axis (Fig. 3*A*).

Anterior coracoid height (ACH) was defined analogously as the perpendicular distance from the glenoid center to the most inferior cortical point of the coracoid, measured orthogonally to the same reference axis (Fig. 3*B*).

#### Reliability

Interobserver and intraobserver reliability were assessed in a random subset of radiographs using intraclass correlation coefficients (ICCs; two-way random-effects model, absolute agreement). Measurements were repeated after a minimum interval of two weeks to minimize recall bias.

There were no missing data for PAC, ACC, GRC, PAH, or ACH in the final cohort.

#### Computed tomography evaluation

In shoulders with pre-operative CT, glenoid version and static PHHS were obtained from three-dimensional planning software (version 4.2.2; Blueprint, Stryker), which automatically calculates these parameters.

### Statistical analysis

The primary analyses compared PAC between Neutral and Walch B shoulders and ACC between Neutral and Latarjet shoulders. Continuous variables were assessed for normality using the Shapiro-Wilk test. As normality assumptions were violated, two-sided Mann-Whitney *U* tests were used for between-group comparisons. Data are reported as median and interquartile range (IQR), with effect size reported as r (Z/√N).

*P* values for related morphometric variables were adjusted using the Benjamini-Hochberg false discovery rate (FDR) procedure (α = 0.05). All reported *P* values for morphometric between-group comparisons are false discovery rate-adjusted. Logistic regression analyses were performed to evaluate associations between morphometric parameters and group allocation. For osteoarthritic shoulders, a multivariable model included PAC, glenoid retroversion, and PHHS. For anterior instability, a univariable model assessed ACC. Results are presented as odds ratios (ORs) with 95% confidence intervals (CIs).

Receiver operating characteristic analysis was performed to evaluate the discriminative ability of PAC and ACC to distinguish between the predefined study groups. Area under the curve (AUC), 95% CIs, and exploratory Youden-derived cutoff values were reported. Interobserver and intraobserver reliability were assessed using a two-way random effects ICC with absolute agreement [ICC (2, 1)].

All statistical analyses were performed using R statistical software (version 4.1.2; R Foundation for Statistical Computing, Vienna, Austria).

## Results

### Patient demographics

A total of 150 shoulders were included: 50 Neutral, 50 Walch B, and 50 Latarjet (Table I). Neutral and Walch B represented older patients with glenohumeral osteoarthritis, whereas the Latarjet cohort comprised substantially younger, predominantly male patients with recurrent anterior instability. Glenoid retroversion and PHHS differed between Neutral and Walch B, as expected from the underlying pathology.

### Posterior acromial coverage

**Figure 4:**
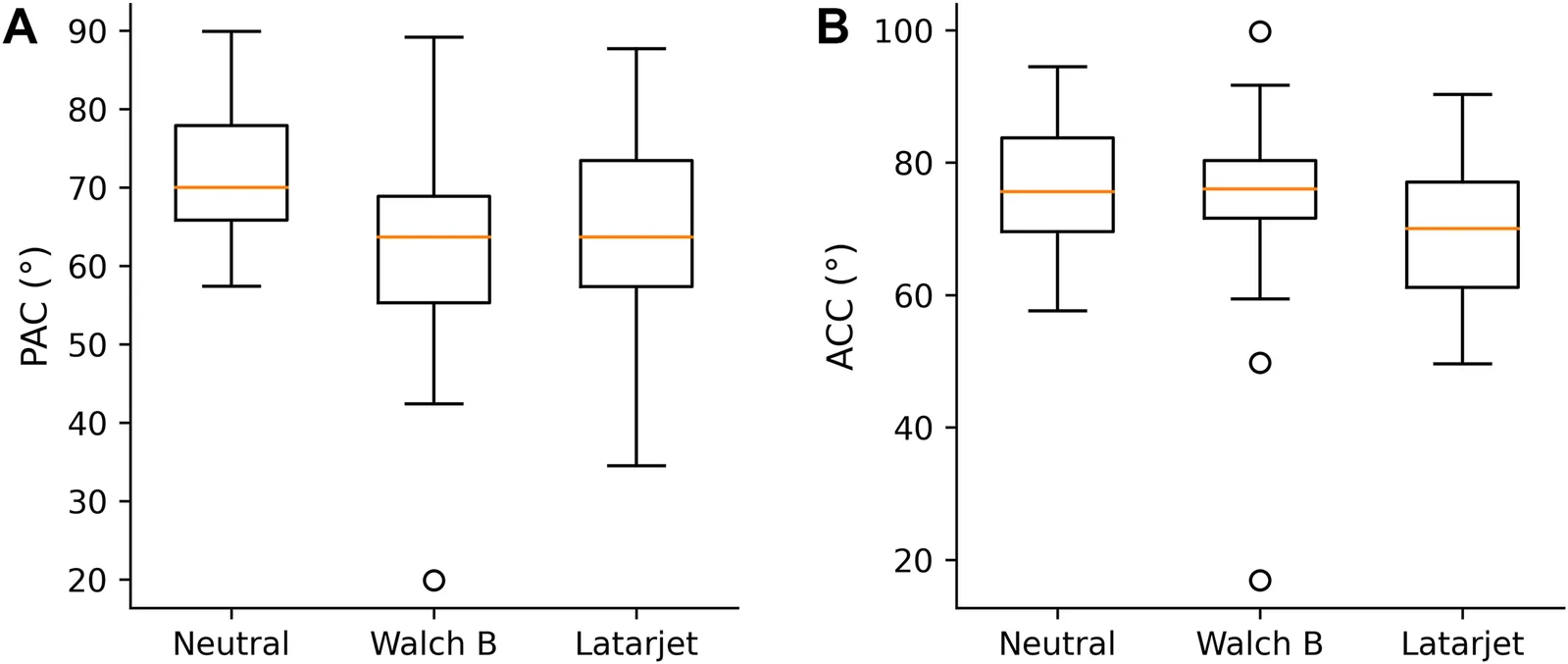
Boxplots of PAC and ACC across cohorts. PAC (**A**) and ACC (**B**) distributions are shown for Neutral, Walch B, and Latarjet. Boxes represent the interquartile range, horizontal lines the median, and whiskers the range excluding outliers. *PAC*, posterior acromial coverage; *ACC*, anterior coracoid coverage.

**Figure 5:**
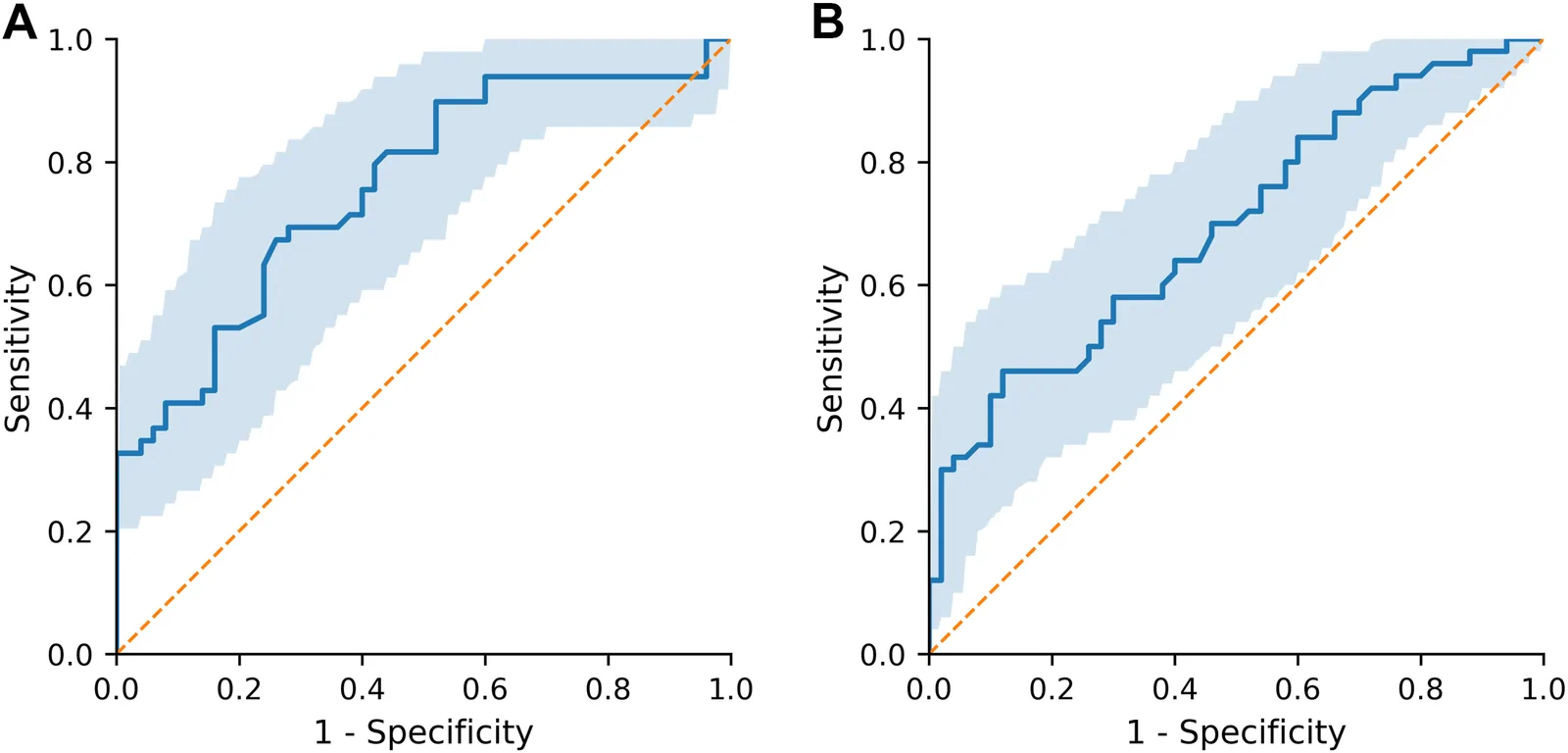
Receiver operating characteristic (ROC) analysis of directional roof parameters. (**A**) ROC curve of posterior acromial coverage (PAC) to discriminate Walch B from Neutral shoulders (AUC 0.76; 95% CI 0.66–0.85). The optimal Youden-derived cutoff was ≤66.2°, corresponding to 69% sensitivity and 72% specificity. (**B**) ROC curve of anterior coracoid coverage (ACC) to discriminate Latarjet from Neutral shoulders (AUC 0.70; 95% CI 0.59–0.79). The optimal Youden-derived cutoff was ≤67.7°, corresponding to 46% sensitivity and 88% specificity. *AUC*, area under the curve; *CI*, confidence interval.

PAC was lower in Walch B than in Neutral shoulders (63.2° [55.3-68.9] vs. 70.0° [65.9-77.9]; *P* < .001, r = −0.52) (Fig. 4*A*; Table II). As expected, Walch B also showed greater PHHS (71.0% [67.0-77.0] vs. 54.0% [46.8-62.8]; *P* < .001, r = 0.84) and increased glenoid retroversion (11.0° [9.0-16.0] vs. 4.0° [3.0-7.0]; *P* < .001, r = 0.83). PAC moderately discriminated Walch B from Neutral (AUC 0.76, 95% CI 0.66-0.85; cutoff ≤66.2° with 69% sensitivity and 72% specificity) (Fig. 5*A*; Table III).

**Table II:** Morphometric parameters by group.

Variable	Neutral (n = 50) Median [IQR]	Walch B (n = 50) Median [IQR]	Latarjet (n = 50) Median [IQR]	Neutral vs. Walch B (*P*)∗	Neutral vs. Latarjet (*P*)∗	Walch B vs. Latarjet (*P*)∗
GRC (°)	146.6 [138.5-156.6]	139.8 [128.2-147.9]	131.9 [120.4-148.5]	.006	<.001	.189
PAC (°)	70.0 [65.9-77.9]	63.2 [55.3-68.9]	63.7 [57.4-73.5]	<.001	<.001	.482
ACC (°)	75.6 [69.6-83.7]	76.0 [71.6-80.3]	70.1 [61.2-77.1]	.905	.001	.003
ACH (mm)	10.8 [5.7-14.4]	11.2 [7.6-16.3]	15.1 [9.3-22.9]	.582	<.001	.005
PAH (mm)	13.3 [9.7-19.2]	18.1 [13.6-24.4]	20.4 [14.2-24.6]	.001	.001	.560

*PAC*, posterior acromial coverage; *ACC*, anterior coracoid coverage; *GRC*, glenoid roof coverage; *PAH*, posterior acromial height; *ACH*, anterior coracoid height; *IQR*, interquartile range.

∗ *P* values were adjusted using the Benjamini-Hochberg false discovery rate procedure.

**Table III:** ROC parameters by group.

Contrast	Marker	Cut-off rule	Sensitivity	Specificity	AUC (95% CI)
Latarjet vs. Neutral	ACC	ACC ≤67.7°	46%	88%	0.696 [0.586-0.794]
Walch B vs. Neutral	PAC	PAC ≤66.2°	69%	72%	0.760 [0.659-0.851]

*PAC*, posterior acromial coverage; *ACC*, anterior coracoid coverage; *AUC*, area under the curve; *ROC*, receiver operating characteristic; *CI*, confidence interval.

### Anterior coracoid coverage

ACC was lower in Latarjet than in Neutral (70.1° [61.2-77.1] vs. 75.6° [69.6-83.7]; *P* = .001, r = −0.39) (Fig. 4*B*; Table II). ACC did not differ between Neutral and Walch B (76.0° [71.6-80.3]; *P* = .905, r = −0.14) but was also lower in Latarjet than in Walch B (*P* = .003, r = −0.34). ACC moderately discriminated Latarjet from Neutral (AUC 0.70, 95% CI 0.59-0.79; cutoff ≤67.7° with 46% sensitivity and 88% specificity) (Fig. 5*B*; Table III).

### Glenoid roof coverage

GRC was highest in Neutral (146.6° [138.5-156.6]) and reduced in Walch B (139.8° [128.2-147.9]) and Latarjet (131.9° [120.4-148.5]). Differences were significant for Neutral versus Walch B (*P* = .006, r = −0.32) and Neutral versus Latarjet (*P* < .001, r = −0.46) but not between Walch B and Latarjet (*P* = .19).

### Vertical parameters

PAH was lowest in Neutral shoulders (13.3 mm [9.7-19.2]) and significantly higher in Walch B (18.1 mm [13.6-24.4]; *P* = .001, r = 0.39) and Latarjet (20.4 mm [14.2-24.6]; *P* = .001, r = 0.39). There was no relevant difference between Walch B and Latarjet (*P* = .560, r = 0.07). ACH was significantly higher in Latarjet (15.1 mm [9.3-22.9]) compared with Neutral (10.8 mm [5.7-14.4]; *P* < .001, r = 0.41) and Walch B (11.2 mm [7.6-16.3]; *P* = .005, r = 0.33), whereas Neutral and Walch B did not differ (*P* = .582, r = 0.06).

### Interobserver reliability

Interobserver reliability was high for all measurements: PAC ICC 0.85 (95% CI 0.75-0.92), ACC ICC 0.90 (95% CI 0.81-0.93), PAH ICC 0.86 (95% CI 0.75-0.92), and ACH ICC 0.88 (95% CI 0.76-0.94).

### Intraobserver reliability

Intraobserver reliability was similarly high across all measurements: PAC ICC 0.89 (95% CI 0.80-0.94), ACC ICC 0.92 (95% CI 0.85-0.96), PAH ICC 0.88 (95% CI 0.79-0.93), and ACH ICC 0.90 (95% CI 0.83-0.95).

### Logistic regression analysis

In the osteoarthritic cohorts, lower PAC was associated with Walch B morphology in univariable logistic regression (OR 0.90 per degree, 95% CI 0.85-0.95; *P* < .001; Table IV). In a multivariable model including PAC, glenoid retroversion, and PHHS, glenoid retroversion (OR 1.34 per degree, 95% CI 1.06-1.69; *P* = .015) and PHHS (OR 1.16 per percent, 95% CI 1.05-1.29; *P* = .005) remained independently associated with Walch B morphology, whereas PAC did not retain an independent association. This attenuation of the PAC association after adjustment indicates collinearity with established three-dimensional parameters of posterior decentering.

**Table IV:** Logistic regression analyses for group allocation.

Explanatory variables	Univariable logistic regression		Multivariable logistic regression	
	OR (95% CI)	*P* value	OR (95% CI)	*P* value
Latarjet vs. Neutral as the dependent variable				
ACC	0.92 (0.88-0.96)	<.001	Not applicable	Not applicable
Walch B vs. Neutral as the dependent variable				
PAC	0.90 (0.85-0.95)	<.001	0.95 (0.88-1.03)	.22
Glenoid retroversion	Not applicable	Not applicable	1.34 (1.06-1.69)	.015
PHHS	Not applicable	Not applicable	1.16 (1.05-1.29)	.005

*PHHS*, posterior humeral head subluxation; *PAC*, posterior acromial coverage; *ACC*, anterior coracoid coverage; *CI*, confidence interval; *OR*, odds ratio.

For anterior instability, lower ACC was associated with Latarjet morphology in univariable logistic regression (OR 0.92 per degree, 95% CI 0.88-0.96; *P* < .001). In exploratory models adjusted for age, sex, and generalized hyperlaxity, the direction and magnitude of the ACC association were similar; however, due to numerical instability related to quasi-complete separation, these multivariable estimates are not reported.

## Discussion

This study demonstrated distinct patterns of superior glenoid roof morphology across Neutral, Walch B, and Latarjet. PAC was selectively reduced in posteriorly decentered shoulders, whereas ACC was selectively reduced in shoulders with chronic anterior instability treated with a Latarjet procedure. These associations were direction-specific and reproducible, with excellent interobserver reliability. The similarly high intraobserver reliability across all angular and vertical parameters further supports the robustness and reproducibility of these radiographic measurements. Together with differences in GRC, these findings support the use of simple Neer Y-view–based morphometric parameters to characterize anterior and posterior roof morphology.^4^^,^^6^^,^^10^^,^^12^^,^^15^^,^^18^^,^^19^

### Posterior instability and posterior acromial coverage

Neutral and Walch B shoulders in this cohort were of comparable age and represent different morphologic expressions within the degenerative spectrum. Therefore, age-related scapular remodeling alone is unlikely to account for the observed differences in roof geometry. Degenerative changes on the posterior acromion are generally uncommon. Walch B shoulders showed a selective reduction in PAC, with values approximately 7° lower than in Neutral shoulders, together with increased glenoid retroversion and PHHS.^12^^,^^16^ These findings confirm the primary hypothesis that PAC is reduced in posteriorly decentered shoulders. As previously reported, PAC reflects the posterior contour of the coracoacromial arch that interacts with horizontally oriented rotator cuff forces acting on the glenoid.^7^^,^^14^ Alterations of this contour may reduce posterior bony containment and facilitate posterior translation or decentering. In Walch B arthropathy, the combination of reduced PAC and increased glenoid retroversion suggests a posterior roof deficiency that is not fully captured by glenoid version alone. These observations are consistent with biomechanical and morphologic studies highlighting the role of acromial anatomy in posterior shoulder stability.^10^^,^^12^^,^^15^^,^^17^ This finding suggests that PAC is not an independent predictor of Walch B morphology but a radiographic expression of posterior decentering, reflecting a secondary morphologic feature rather than an independent driver once established three-dimensional parameters such as glenoid retroversion and PHHS are considered. The attenuation after adjustment suggests that PAC represents a radiographic manifestation of posterior structural imbalance already quantified by three-dimensional parameters. As such, PAC provides an accessible two-dimensional surrogate of posterior roof configuration on routine radiographs. PAC should therefore not be viewed as redundant but as a projection-based radiographic expression of posterior structural imbalance that can be identified before advanced imaging is performed.

### Anterior instability and anterior coracoid coverage

The Latarjet cohort consisted of young patients with chronic traumatic anterior instability and structurally unstable joints. In this group, ACC was selectively reduced compared with Neutral, whereas ACC did not differ between Neutral and Walch B shoulders. This pattern indicates that reduced ACC is associated with chronic anterior instability. Age-related changes in coracoid or posterior acromion morphology are highly unlikely in this group. The Neutral cohort consists of arthritic shoulders with neutral glenoid alignment and therefore represents a clinically stable reference group, with no documented instability episodes despite long-term physiological loading. This distinguishes it from young asymptomatic control populations, in whom future instability cannot be excluded. Within this context, reduced ACC reflects diminished anterior bony containment of the humeral head once capsulolabral restraints are compromised. These findings extend previous observations on coracoacromial morphology and anterior instability to a simple radiographic metric measurable on standard Y-views.^12^^,^^16^ ACC showed moderate diagnostic discrimination, with high specificity but limited sensitivity and should therefore be regarded as a promising morphologic descriptor rather than a clinical decision tool.

### Global roof coverage

GRC, as an integrated measure of anterior and posterior roof width, was highest in Neutral shoulders and reduced in both Walch B and Latarjet. There was no significant difference in GRC between Walch B and Latarjet, suggesting that the pattern of roof deficiency is more informative than the absolute global width. As GRC is calculated as the sum of PAC and ACC, it is mathematically dependent on both parameters and therefore it does not represent an independent variable.

In addition to the angular roof parameters, the vertical measurements demonstrated complementary patterns. PAH was lowest in Neutral and increased in both Walch B and Latarjet, whereas ACH was selectively increased in Latarjet. These findings are consistent with prior investigations demonstrating associations between posterior acromial morphology and posterior instability^10^^,^^15^^,^^17^ as well as between coracoacromial arch configuration and anterior instability.^12^^,^^16^

Walch B shoulders showed reduced PAC with preserved ACC, whereas Latarjet shoulders showed reductions in both parameters but more pronounced anteriorly, consistent with a more global roof undercoverage.

This constellation is compatible with recent experimental observations reporting altered humeral head pathokinematics after coracoid transfer procedures, including posteriorly biased translation under unloaded conditions.^7^^,^^13^^,^^14^ However, given the cross-sectional design of the present study, these associations should be interpreted descriptively rather than causally.

## Clinical implications

The moderate discriminative performance observed in receiver operating characteristic analyses demonstrates that PAC and ACC meaningfully distinguish the predefined study groups and supports their use as structured radiographic morphometric descriptors rather than independent diagnostic classifiers. The derived thresholds are hypothesis-generating and not intended for individual surgical decision-making. Based on standardized Neer lateral Y-view radiographs, these parameters provide reproducible and accessible measures of superior glenoid roof geometry for routine assessment.

In posteriorly decentered shoulders with moderate glenoid retroversion, reduced PAC may indicate a posteriorly oriented roof configuration not fully captured by glenoid version or humeral head subluxation alone. In such cases, PAC may support identification of patients who warrant advanced three-dimensional imaging or further structural evaluation. Conversely, in young patients with recurrent anterior instability and limited glenoid bone loss, reduced ACC may reflect diminished anterior bony containment of the humeral head and subscapularis and provide additional morphologic context.

PAC and ACC represent reproducible radiographic components of instability direction-specific roof morphology and enable recognition of reduced coverage in clinical practice prior to advanced imaging. However, external validation is required before any clinical application.

## Limitations

This single-center study with retrospective radiographic analyses may be subject to selection bias, and residual confounding related to age, sex, or activity level. Chronic remodeling of the coracoid and posterior acromion is unlikely in the 3 cohorts but cannot be excluded. The posterior acromial contour and coracoid morphology are not typically subject to degenerative shape changes, reducing the likelihood that age alone explains the observed differences. The Neutral cohort consists of elderly patients with glenohumeral osteoarthritis undergoing arthroplasty and must therefore be interpreted as a clinically stable arthritic reference group rather than as a representation of normal scapular morphology. We acknowledge that the absence of a younger asymptomatic control group limits generalizability, and the reported values should not be regarded as normal. All measurements were derived from two-dimensional radiographs and therefore simplify three-dimensional anatomy; external validation against advanced imaging and biomechanical models is required. None of the 3 parameters was correlated with recurrence, failure, patient-reported outcomes, instability severity, or surgical outcome. Therefore, clinical validation is required before application of PAC, ACC, and GRC in decision-making. Finally, the cross-sectional design precludes conclusions regarding causality or clinical outcomes.

## Conclusion

Compared with a clinically stable arthritic reference cohort, static posterior subluxation (Walch B) was associated with reduced PAC, whereas chronic anterior instability was associated with reduced ACC. GRC was reduced in both pathologic groups, consistent with the concept of direction-specific superior glenoid roof morphology. PAC and ACC provide reproducible radiographic quantification of posterior and anterior bony containment of the humeral head on routine Neer lateral Y-view radiographs. Integration of superior roof geometry into standard radiographic assessment provides a reproducible radiographic framework for recognizing direction-specific patterns of shoulder instability. As association with clinical outcomes was not assessed, these parameters require further clinical and external validation.

## Disclaimers:

Funding: No funding was disclosed by the authors.

Conflicts of interest: William G. Blakeney is a paid consultant for Enovis/Lima, and Stefan Bauer is a paid consultant for Stryker and Enovis. Any additional authors, their immediate families, and any research foundations with which they are affiliated have not received any financial payments or other benefits from any commercial entity related to the subject of this article.
